# Exploring Continuum and Categorical Conceptualisations of Mental Health and Mental Illness on Australian Websites: A Systematic Review and Content Analysis

**DOI:** 10.1007/s10597-022-01005-w

**Published:** 2022-08-22

**Authors:** Dominic K. Fernandez, Saniya Singh, Frank P. Deane, Stewart A. Vella

**Affiliations:** School of Psychology, University of Wollongong, North Wollongong, NSW 2522 Australia

**Keywords:** Continuum conceptualisation, Categorical conceptualisation, Cause; systematic review, Mental health, Mental illness

## Abstract

It is important to explore the types of conceptualisations and causes presented in online mental health promotion given the implications that these presentations may have on mental health stigma. This study systematically reviewed 92 Australian webpages focused on either mental health, mental illness, depression, or schizophrenia, to explore the types of conceptualisations and aetiologies presented. A minority of mental health and mental illness webpages (*n* = 8, 8.70%) explicitly presented continuum conceptualisations, with none providing explicit categorical conceptualisations. No depression or schizophrenia webpages presented explicit conceptualisations of any kind. All four webpage foci had a greater proportion of continuum than categorical conceptualisations. Moreover, both depression and schizophrenia webpages presented many mixed conceptualisations which included both continuum and categorical messaging. Most webpages mentioned biological and social causes equally across webpage foci. These findings suggest that Australian mental health websites predominantly present continuum conceptualisations of mental health and mental illness.

**Supplementary Information:**

The online version contains supplementary material available at 10.1007/s10597-022-01005-w.

## Introduction

### Online Mental Health Information

The internet is an increasingly important medium through which the general public can access mental health information (Hollis et al., 2015). As of 2017, 87% of Australians aged 15 years or over had used the internet at least once in the past 3 months (Australian Bureau of Statistics, 2018). These numbers are even higher for young Australians aged 15–24 years, with between 97 and 98% of them accessing the internet in 2016–2017 (Australian Bureau of Statistics, 2018). Moreover, over half of young people aged 16–25 years who are experiencing psychological distress use the internet to seek information about mental health (Burns et al., 2016). Given the near ubiquity of internet usage in Australia, websites that provide information about mental health and mental illness are uniquely positioned to educate large numbers of Australians about these topics. For example, there were almost 13 million visitors to the Beyond Blue website in a single year (Beyond Blue, 2019).

However, there are limitations to disseminating mental health information online. Firstly, any person or organisation can publish material about mental health online. While there are indicators of online health information quality, no formal body oversees the quality of these websites (Grohol et al., 2013). Furthermore, research has found that websites which receive funding from pharmaceutical companies are significantly more likely to present biogenetic aetiologies of mental illness which support their commercial interests compared to unaffiliated websites (Read & Cain, 2013). As such, it is important to investigate how prominent websites present mental health and mental illness, including how they conceptualise these phenomena and the causes they ascribe to them.

### Stigma

Stigma is a process through which individuals are discriminated against, devalued or discredited based on personal characteristics (Weiss et al., 2006). The discrimination experienced as a result of mental health stigma among the general public reduces quality of life (Switaj et al., 2009), self-esteem and hope of recovery (Wahl, 1999; Yanos et al., 2008), educational attainment (Van Brakel, 2006), employment prospects, and social functioning (Sayce, 2000). Given this, mental health stigma has become a prominent policy target for improving public mental health outcomes (Commonwealth of Australia, 2009). Despite considerable research into stigma reduction (Griffiths et al., 2014; Morgan et al., 2018) and efforts in mental health promotion (Jorm, 2012), stigmatising attitudes towards mental illness have remained fairly stable over the past 3 decades (Schomerus et al., 2012). This is despite noted improvements to mental health literacy (Angermeyer & Matschinger, 2005; Jorm et al., 2005a, 2006). Therefore, it is important to ensure that mental health promotion is informed by the stigma reduction literature.

### Conceptualisations of Mental Health and Mental Illness

One important factor influencing mental health stigma is the way in which mental illness is conceptualised (Kvaale et al., 2013; Schomerus et al., 2016). The biomedical, sometimes known as biogenetic, conceptualisation of mental illness has increasingly become the dominant explanatory account of psychopathology (Lebowitz & Appelbaum, 2019; Pescosolido et al., 2010). This conceptualisation views mental illness as a medical illness caused by biological and genetic factors that affect normal brain functioning (Deacon, 2013). It is typically associated with a categorical conceptualisation of mental illness as it often suggests that there are qualitative differences between having a mental illness and not having one. Part of the appeal of this conceptualisation is that it reduces the stigmatising attitude of blame towards people with mental illness (Kvaale et al., 2013). However, endorsement of the biogenetic causes underpinning the biomedical model has also been found to increase prognostic pessimism and perceived dangerousness without influencing social distance (Kvaale et al., 2013), an individual’s unwillingness to engage with people with mental illnesses. Given the shortcomings of this conceptualisation, recent research has explored the continuum conceptualisation of mental illness as a potential alternative.

A recent systematic review and meta-analysis by Peter et al. (2021) found that continuum beliefs about mental illness are associated with less stigmatising attitudes (Schomerus et al., 2013; Subramaniam et al., 2017; Wiesjahn et al., 2014). However, their review of the experimental findings was somewhat mixed. While some research indicated continuum belief manipulations are beneficial at reducing stigma (Cole & Warman, 2019; Corrigan et al., 2017; Schomerus et al., 2016), other research found no effect of continuum belief manipulations on stigma (Thibodeau, 2017, 2019), or even adverse effects such as increased fear (Thibodeau & Peterson, 2018). Thus, while the evidence supporting the efficacy of continuum conceptualisations as a stigma reduction strategy is still emerging, Peter et al. (2021) concluded that continuum belief explanations should be included in anti-stigma campaigns as a promising stigma reduction strategy.

This increase in research into the stigma-reducing potential of continuum conceptualisations follows substantial criticism of the categorial nosology of mental illness found in the DSM-IV (Brown & Barlow, 2005; Krueger & Bezdjian, 2009) and a subsequent small shift towards integrating a dimensional approach within the DSM-V (Regier et al., 2013), particularly for personality disorders. Further, stigma experts recommend against categorical conceptualisations within mental health promotion campaigns (Clement et al., 2010).

Researchers have suggested that mental illnesses do not fall neatly into distinct categories, but rather share underlying dimensions and processes (Forgeard et al., 2011). For example, there is empirical support for the continuum nature of psychotic symptoms (DeRosse & Karlsgodt, 2015; van Os et al., 2009) and depressive symptoms (Ayuso-Mateos et al., 2010). Haslam et al. (2020) conducted a systematic review and meta-analysis examining the taxometric evidence for dimensional and categorical models of various psychological constructs. They found that mood disorders (e.g., depression) were consistently dimensional in nature. Further, the findings for psychotic disorders (e.g., schizophrenia) also favoured dimensionality but were inconclusive. Importantly, neither psychopathology nor regular psychological variation were found to fit the categorical model. That is, the underlying structure of psychopathology is no more amenable to being categorised than general psychological functioning is. Moreover, another review by Iasiello et al. (2020) has shown that mental health and mental illness make up separate but related continua. Given the concerns regarding the effect of categorical conceptualisations of mental illness on stigma, in addition to the growing empirical evidence for dimensional models, it is important to investigate how mental illness and mental health are being conceptualised in real world mental health promotion.

Despite these concerns about categorical conceptualisations of mental illness, a recent systematic review examining YouTube videos about depression found that categorical conceptualisations are much more likely to be presented than continuum conceptualisations (Devendorf et al., 2020). This is understandable given the growing acceptance of the biomedical conceptualisation of mental illness among both the general public (Schomerus et al., 2012) and mental health and medical organisations (Deacon, 2013). While biomedical conceptualisations do not explicitly endorse a categorical conceptualisation, they provide implicit support through promoting essentialism (Lebowitz & Appelbaum, 2019), the belief that members within a group share an underlying ‘essence’ (Medin & Ortony, 1989). Haslam and Kvaale (2015) have also suggested that biogenetic explanations of mental illness, which fall within the biomedical model, promote essentialist thinking. Essentialism has been linked with stigmatising attitudes (Haslam & Whelan, 2008) and stereotyping (Bastian & Haslam, 2006). However, Devendorf et al. (2020) did not find any significant difference between the presentations of biological or environmental aetiologies. This is not unexpected, as endorsement of psychosocial causes by the general public remains high despite the increasing acceptance of biological causes (Schomerus et al., 2012). Moreover, the preponderance of categorical conceptualisations was in part due to a decision to code all videos that did not mention a continuum conceptualisation as categorical (Devendorf et al., 2020). As such, it is unclear what proportion of videos coded as categorical actually presented a categorical conceptualisation, either explicitly or implicitly. Research is needed to examine online mental health and mental illness conceptualisations in a more fine-grained manner to provide a richer understanding of the ways in which conceptualisations of mental health and mental illnesses are presented online.

### Aims

There is a dearth of literature exploring how leading mental health organisations conceptualise mental health and mental illness in online settings. Given the potential impacts that different conceptualisations can have on mental health stigma, it is important to investigate how Australian online mental health organisations conceptualise mental health and mental illness. The primary aim of this review is to explore the prevalence of continuum and categorical conceptualisations of mental health and mental illness within Australian online mental health promotion. Specifically, we will investigate mental health, mental illness, schizophrenia, and depression. These foci were chosen to both explore how mental health and illness in general are conceptualised, as well as to see how less-stigmatised mental illnesses such as depression are conceptualised relative to less common, more stigmatised mental illnesses, like schizophrenia (Reavley & Jorm, 2011). Furthermore, Australian websites were chosen as Australia’s disease burden attributable to mental disorders is among the highest globally, according to the World Health Organization’s Global Burden of Disease Study (2022). A secondary aim of this review was to investigate the prevalence of proposed causes of these mental illnesses to explore whether biological causes were more prevalent than social causes.

## Theory

### Theoretical and Conceptual Framework

Our theoretical framework draws from Link and Phelan’s (2001) Modified Labelling Theory, Corrigan’s (2004) model of public and self-stigma, Leventhal et al.’s (1984) Common Sense Model of illness representations (CSM), and Devendorf et al.’s (2020) conceptual framework for depression. Link and Phelan (2001) posit that stigmatisation occurs when four components are present: (1) labelling based on perceived differences, (2) linking labels to negative stereotypes, (3) categorisation into in-group and out-group, (4) status loss and discrimination. The CSM proposes that an individual’s mental representations about mental illness are drawn from three sources of information: lay information from social communication and cultural knowledge (e.g., websites), external informal and professional sources (e.g., friends, psychologists), and direct exposure via having or knowing someone who has a mental illness (e.g., having experienced schizophrenia). While mental representations include various dimensions such as causes, consequences, and timeline (Hagger & Orbell, 2003), our theoretical framework focuses on the dimension of conceptualisation (continuum vs. categorical).

**Fig. 1:**
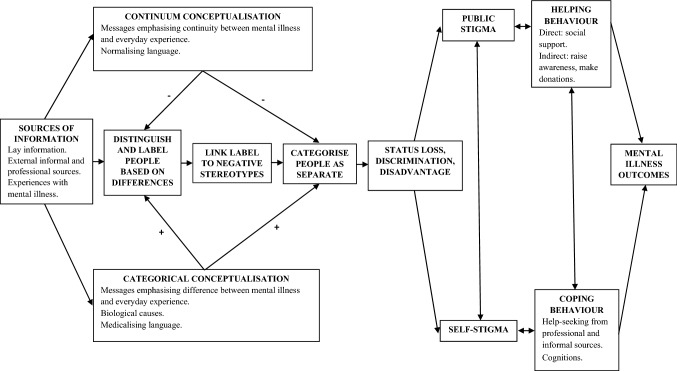
Conceptual framework

These theories informed the development of a similar conceptual framework to Devendorf et al., (2020), with the notable addition of Link and Phelan’s (2001) stigmatisation process (see Fig. 1). This framework illustrates the pathways through which sources of information (e.g., mental health websites) may impact the stigmatisation process via conceptualisations, which in turn influence helping and coping behaviours, ultimately affecting mental illness outcomes. We propose that continuum conceptualisations may reduce stigma by reducing perceptions of difference and dissolving in-group and out-group categories. Meanwhile, the opposite pattern is proposed for categorical conceptualisations.

## Methods

### Website Search and Selection

The first author conducted keyword searches on Google.com.au using Incognito mode on March 9th and May 15th, 2020, in Australia. An additional search was conducted on May 21st, 2021, from which 10 additional webpages were added. All included webpages were also re-examined at this time to ensure the information was up to date, at which stage one previously included webpage was removed as it no longer existed. As of April 2022, Google made-up 93.75% of all web searches in Australia (Statcounter Global Stats, 2022). Therefore, Google was searched for the following keywords: “mental health”, “what is mental health”, “mental health Australia”, “mental illness”, “what is mental illness”, “mental illness Australia”, “depression”, “depressive disorders”, “what is depression”, “depression Australia”, “schizophrenia”, “what is schizophrenia”, and “schizophrenia Australia”. A final search was conducted on May 10th, 2022, to update webpage information and search for new webpages. This search included the additional search terms: “mental disorder”, “what is mental disorder”, “mental disorder Australia”, “mental disease”, “what is mental disease”, and “mental disease Australia”. These additional terms were included to ensure that biomedical perspectives were not underrepresented. Their inclusion led to the identification of a single additional webpage which was added to the review.

In line with earlier research (Zermatten et al., 2010), only the first 20 websites were examined from each search as people rarely search beyond the first 20 links (Eysenbach et al., 2002). Furthermore, the snowball method was used where links to other websites provided by eligible websites were assessed for eligibility, as were additional webpages within a given website. Websites were excluded if they were: not Australian, inaccessible (broken link), news articles, reports, research articles, books, standalone videos, required a login or fee, had no content relevant to the search (e.g., no depression information for depression query), not in English, or were solely an online forum or message board.

**Fig. 2:**
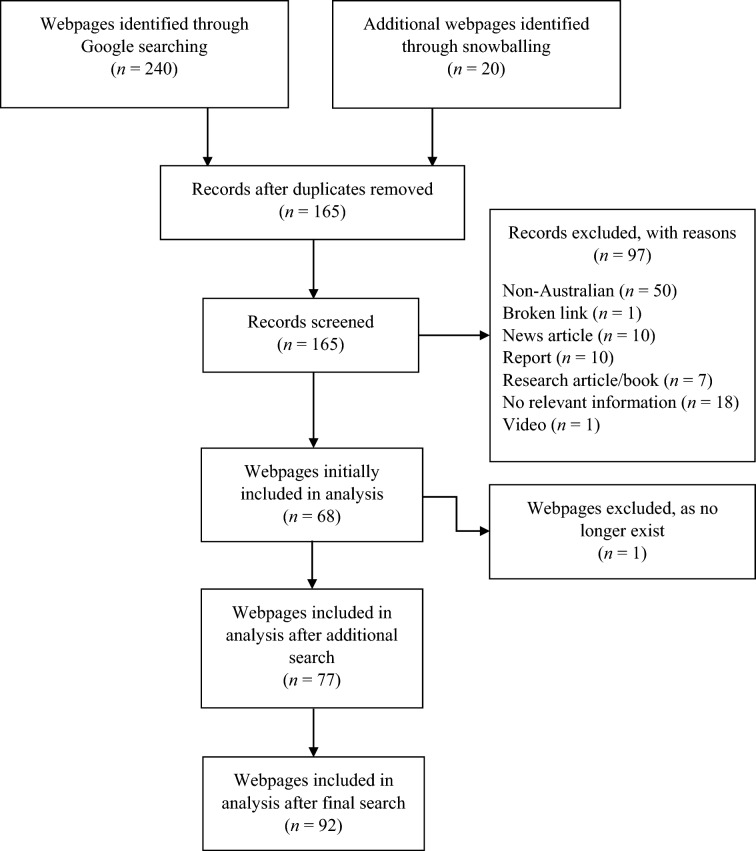
Flowchart of search results

The first author screened all websites and extracted the relevant webpages for coding. See Fig. 2 for webpage search results. Different webpages within a website that had the same focus (e.g., depression) were considered duplicates and combined for analysis. Webpages within a website that had different focuses were not considered duplicates. Likewise, if webpages from the same website had the same focus but were targeted at a different population (e.g., young people vs. general population), these were also considered different webpages.

### Webpage Coding

The first author coded all 92 webpages using the coding criteria (see Supplementary materials A). Webpages were coded for the following information:

1. Webpage focus: The four foci of mental health, mental illness, depression, and schizophrenia were assessed from the webpage titles and content. Mental health was divided into 2 sub-categories, ‘good wellbeing’, and ‘general wellbeing’, as piloting of the coding criteria indicated that mental health was defined in two disparate ways. A good wellbeing focus was present when mental health was defined as a positive state (e.g., being able to cope with stresses and contribute to one’s community). Meanwhile, a general wellbeing focus defined mental health as inclusive of both good and poor wellbeing.
2. Target population: Ascertained from the webpage itself, or other sections of the website such as the ‘about us’ page.
3. Medicalisation: Assessed by examining whether the webpage referred to mental illness, depression, or schizophrenia as being a medical illness (e.g., “Depression is a real illness”). Use of the terms ‘mental illness’ or ‘mental disorder’ on their own were not considered sufficient to characterise the conceptualisation as medicalising given the widespread usage of these terms.
4. Conceptualisation: The webpage was examined to investigate whether they explicitly (e.g., “mental health is on a continuum”) or implicitly (e.g., “people’s mental health varies from good mental health to poor mental health”) conceptualised mental health or mental illnesses as being on a continuum, categorical, or some combination of the two. Moreover, the data were further coded for whether the continuum conceptualisations were within mental health and mental illness (e.g., from mild to severe mental illness), and/or between mental health and mental illness (e.g., from good mental health to severe mental illness). Likewise, the implicit categorical conceptualisations were further coded as either medicalising (e.g., “depression is a serious illness”) and/or implying a difference (e.g., “depression is very different from everyday sadness”).
5. Causes: Coding criteria for the causes of mental illness were in part derived from the Mental Illness Attribution Questionnaire (Knettel, 2019). Proposed causes were coded into the following categories: biological, social, psychological, biosocial, biopsychosocial, lifestyle, personal weakness, substance use, personality. Biosocial and biopsychosocial causes were only labelled when pages explicitly mentioned the interplay of different causes.
6. Health on the Net Badge: Each webpage URL was entered into the Health on the Net HONcode search tool to explore whether it was a certified page. Health on the Net certification indicates reliable and transparent online health information.
7. Affiliation: The affiliation of websites was categorised as: commercial, non-profit, government, university, or personal.
8. Coverage: Local, state, or national.

The coding criteria allows for mixed conceptualisations since pilot testing of the criteria indicated that some webpages presented information supporting both categorical and continuum conceptualisations. For example, the Way Ahead depression webpage states: “Depression is … significantly different from mere unhappiness or sadness”, which implies a categorical difference. The page goes on to medicalise depression, saying: “It is a long lasting, often recurring illness as real and debilitating as heart disease.” However, it also implies a continuum within depression by explaining: “The symptoms and the severity of feelings of depression may be different for each person”. Therefore, while the webpage predominantly implies a categorical conceptualisation, it also implies a continuum within depression and as such is coded as “Mixed: Implicit continuum (within), implicit categorical (difference/medicalising)”. This coding criteria provides a nuanced representation of how websites conceptualise mental health and mental illness.

Our criteria also allowed for the distinction of a continuum both within mental health and mental illness, and between them. The continuum between mental health and mental illness departs from Westerhof and Keyes’ (2010) Two Continua Model. Their model proposes that mental health and mental illness are on separate, although related, continua. That is, the former continuum includes the presence and absence of mental health, while the latter includes the presence and absence of mental illness. However, pilot coding indicated that some webpages presented mental health as a continuum *between* mental health and mental illness. As such, webpages focused on mental health that explicitly or implicitly mentioned a continuum from mental health to mental illness were coded ‘between’, as were webpages focused on mental illness, depression or schizophrenia, that mentioned a continuum from mental illness symptoms to the everyday experiences of the general population. Conversely, webpages that mentioned a continuum within mental health (e.g., from good to poor wellbeing) or mental illness (e.g., from severe to mild depression) were coded ‘within’. While the continuum-within code better fits Westerhof and Keyes’ (2010) model of mental illness, it only matches part of their continuum, as it does not extend to the absence of mental illness.

**Fig. 3:**
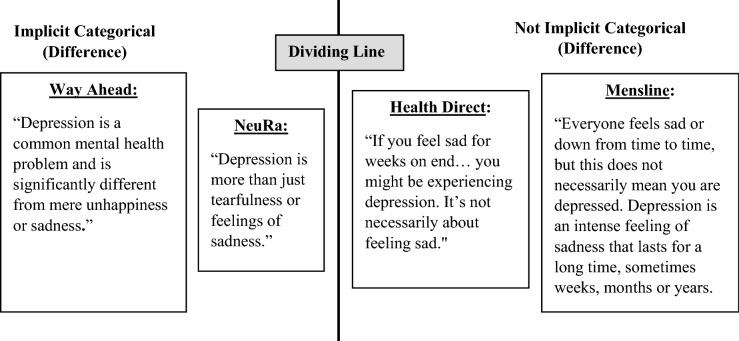
Visualisation of implicit categorical conceptualisation distinctions for depression

There were difficulties with developing coding criteria that clearly delineated between categorical and non-categorical conceptualisations, particularly for depression. For example, our coding criteria define a categorical conceptualisation as a qualitative difference between mental illness and everyday functioning. That is, a difference of ‘kind’, rather than a difference of ‘degree’. Figure 3 below demonstrates how somewhat similar statements can nevertheless differ in terms of whether they meet the threshold for a categorical conceptualisation. The Way Ahead and NeuRa webpages were coded as implying a categorical conceptualisation via presenting a difference whereas the Health Direct and Mensline pages were not. This is because the former two were interpreted as presenting a difference of kind, while the latter two presented a difference of severity or duration. This demonstrates the difficulty in unpacking the type of conceptualisation when webpages are not explicitly stating a particular conceptualisation.

### Webpage Coding Findings

A second coder coded all webpages, including webpage focus, medicalisation, conceptualisation, and causes. Tables 1 and 2 demonstrate at least moderate agreement between coders across all codes, except for lifestyle, which approached moderate agreement. Moderate agreement is represented by a Cohen’s Kappa of 0.41–0.60 (Landis & Koch, 1977) or an intraclass correlation coefficient of 0.50–0.75 (Koo & Li, 2016). Disagreements were resolved by discussion, with all authors consulted where necessary.

**Table 1:** Inter-rater reliability for conceptualisation and medicalisation

Ther	Mental health		Mental illness		Depression		Schizophrenia	
	Agreement (%)	Cohen’s κ	Agreement (%)	Cohen’s κ	Agreement (%)	Cohen’s κ	Agreement (%)	Cohen’s κ
Conceptualisation	72.73	.68, *p* < .001	79.17	.70, *p* < .001	50.00	.44, *p* < .001	68.75	.61, *p* < .001
Medicalisation	–	–	79.17	.68, *p* < .001	90.00	.85, *p* < .001	81.25	.65, *p* < .001

**Table 2:** Inter-rater reliability for webpage focus and causes

Measure	Intraclass correlation	95% C. I		Value	df1	df2	*p*
Total							
Webpage Focus	.86	0.79	0.91	7.38	91	92	< .001
Causes							
Unspecified	.93	0.88	0.95	13.48	69	70	< .001
Biological	.92	0.87	0.95	12.14	69	70	< .001
Social	.93	0.89	0.96	14.99	69	70	< .001
Psychological	.96	0.93	0.97	22.90	69	70	< .001
Biosocial	.78	0.65	0.86	4.60	69	70	< .001
Biopsychosocial	.86	0.77	0.91	6.96	69	70	< .001
Substance Use	.91	0.86	0.95	11.38	69	70	< .001
Personality	.81	0.69	0.88	5.26	69	70	< .001
Lifestyle	.65	0.44	0.78	2.87	69	70	< .001

Personal weakness not included due to neither rater coding this cause

### Declaration of Interests

We declare that this research received funding from an Australian Government Research Training Program Scholarship. The authors have no other conflict of interest to disclose. All authors certify responsibility for the manuscript.

## Results

### Webpage Search

Of the 260 webpages reviewed, only 92 met the criteria for coding and analysis. These webpages were categorised by focus. Of these webpages, there were: 22 mental health webpages (23.91%), 24 mental illness webpages (26.09%), 30 depression webpages (32.61%), and 16 schizophrenia webpages (17.39%).

### Conceptualisation Findings

See Table 3 for a summary of the frequency findings.

**Table 3:** Frequencies for each focus

	Mental health			Mental illness	Depression	Schizophrenia
	Good Wellbeing (*n* = 17)	General Wellbeing (*n* = 6)	Total (*n* = 22)	(*n* = 24)	(*n* = 30)	(*n* = 16)
	Number (%)*	Number (%)*	Number (%)	Number (%)	Number (%)	Number (%)
Conceptualisation						
Continuum	7 (41.18)	5 (83.33)	11 (50.00)	17 (70.83)	29 (96.67)	12 (75.00)
Implicit continuum	4 (23.53)	4 (66.67)	7 (31.82)	16 (66.67)	29 (96.67)	12 (75.00)
Between	1 (5.88)	2 (33.33)	3 (13.64)	3 (12.50)	7 (23.33)	0 (0.00)
Within	3 (17.65)	2 (33.33)	4 (18.18)	12 (50.00)	4 (13.33)	11 (68.75)
Between/Within	0 (0.00)	0 (0.00)	0 (0.00)	1 (4.17)	18 (60.00)	1 (6.25)
Explicit continuum	4 (23.53)	1 (16.67)	5 (22.73)	3 (12.50)	0 (0.00)	0 (0.00)
Between	4 (23.53)	0 (0.00)	4 (18.18)	3 (12.50)	0 (0.00)	0 (0.00)
Within	0 (0.00)	1 (16.67)	1 (4.55)	0 (0.00)	0 (0.00)	0 (0.00)
Between/within	0 (0.00)	0 (0.00)	0 (0.00)	0 (0.00)	0 (0.00)	0 (0.00)
Categorical	1 (5.88)	0 (0.00)	1 (4.55)	10 (41.67)	21 (70.00)	10 (62.50)
Implicit categorical	1 (5.88)	0 (0.00)	1 (4.55)	10 (41.67)	21 (70.00)	10 (62.50)
Difference	1 (5.88)	0 (0.00)	1 (4.55)	2 (8.33)	12 (40.00)	0 (0.00)
Medicalising	0 (0.00)	0 (0.00)	0 (0.00)	7 (29.17)	5 (16.67)	3 (18.75)
Difference/medicalising	0 (0.00)	0 (0.00)	0 (0.00)	1 (4.17)	4 (13.33)	7 (43.75)
Explicit categorical	0 (0.00)	0 (0.00)	0 (0.00)	0 (0.00)	0 (0.00)	0 (0.00)
Unspecified	10 (58.82)	1 (16.67)	11 (50.00)	5 (20.83)	0 (0.00)	4 (25.00)
Mixed	1 (5.88)	0 (0.00)	1 (4.55)	8 (33.33)	20 (66.67)	10 (62.50)
Causes						
Unspecified	–	–	–	11 (45.83)	4 (13.33)	3 (18.75)
Biological	–	–	–	10 (41.67)	26 (86.67)	12 (75.00)
Social	–	–	–	11 (45.83)	26 (86.67)	11 (68.75)
Psychological	–	–	–	5 (20.83)	9 (30.00)	1 (6.25)
Biopsychosocial	–	–	–	5 (20.83)	6 (20.00)	1 (6.25)
Biosocial	–	–	–	3 (12.50)	7 (23.33)	4 (25.00)
Lifestyle	–	–	–	2 (8.33)	5 (16.67)	2 (12.50)
Personality	–	–	–	1 (4.17)	13 (43.33)	0 (0.00)
Substance use	–	–	–	6 (25.00)	12 (40.00)	9 (56.25)
Personal weakness	–	–	–	0 (0.00)	0 (0.00)	0 (0.00)
Medicalising						
Yes	–	–	–	8 (33.33)	9 (30.00)	10 (62.50)
Somewhat	–	–	–	6 (25.00)	6 (20.00)	0 (0.00)
No	–	–	–	10 (41.67)	15 (50.00)	6 (37.50)
Affiliation						
University	1 (5.88)	3 (50.00)	4 (18.18)	0 (0.00)	3 (10.00)	1 (6.25)
Non-Profit	7 (41.18)	2 (33.33)	9 (40.91)	13 (54.17)	19 (63.33)	8 (50.00)
Government	7 (41.18)	0 (0.00)	7 (31.82)	9 (37.50)	8 (26.67)	6 (37.50)
Commercial	2 (11.76)	1 (16.67)	2 (9.09)	2 (8.33)	0 (0.00)	1 (6.25)
Personal	0 (0.00)	0 (0.00)	0 (0.00)	0 (0.00)	0 (0.00)	0 (0.00)
HON^ badge						
Yes	3 (17.65)	1 (16.67)	4 (18.18)	5 (20.83)	7 (23.33)	3 (18.75)
No	14 (82.35)	5 (83.33)	18 (81.82)	19 (79.17)	23 (76.67)	13 (81.25)

^Health on the net

^*^Percentage as a proportion of good wellbeing OR general wellbeing webpages (not percentage of all mental health webpages)

#### Mental Health Conceptualisation

Overall, mental health was much more likely to be conceptualised as a continuum than categorically (*n* = 11, 50.00% vs. *n* = 1, 4.55%). Compared to the other foci, mental health was the least likely to present a mixed conceptualisation (*n* = 1, 4.55%). Moreover, mental health webpages were the most likely to use explicit conceptualisations, with 22.73% (*n* = 5) of webpages presenting an explicit continuum conceptualisation (e.g., Headspace: “Mental health can be thought of as sitting on [a] continuum (or scale) that we all move along, all the time”), compared to 12.50% (*n* = 3) for mental illness, and none for depression or schizophrenia. There were no explicit categorical conceptualisations for any foci, including mental health. Lastly, mental health webpages were the most likely to provide an unspecified conceptualisation (*n* = 11, 50.00%).

Seventeen webpages defined mental health to refer specifically to good wellbeing. Six webpages defined mental health in terms of general wellbeing. Good wellbeing webpages were more likely to present an unspecified conceptualisation than general wellbeing webpages (*n* = 10, 58.82% vs. *n* = 1, 16.67%). While general wellbeing was more likely to be conceptualised as a continuum than good wellbeing (*n* = 5, 83.33% vs. *n* = 7, 41.18%), this difference disappeared when excluding unspecified webpages (100.00% vs. 100.00%). Finally, neither were likely to present a categorical conceptualisation, with only one good wellbeing webpage implying a categorical conceptualisation.

#### Mental Illness Conceptualisation

Mental illness was more likely to be conceptualised as a continuum than categorically (*n* = 17, 70.83% vs. *n* = 10, 41.67%). Of the continuum conceptualisations, the overwhelming majority were implicit (*n* = 16, 94.11%) rather than explicit (*n* = 3, 17.65%). Further, more webpages implied a continuum within mental illness (e.g., Head to Health, “… mental health conditions …can be experienced on a sliding scale from mild to severe, and short-term to longer-term; with some being quite serious.”) than between everyday experience and mental illness (Sydney Local Health District: “While everyone experiences strong feelings of tension, fear, or sadness at times, a mental illness is present when these feelings become so disturbing and overwhelming that people have great difficulty coping…”; *n* = 13, 54.17% vs. *n* = 4, 16.67%).

#### Depression Conceptualisation

While every depression webpage provided a conceptualisation, none were explicitly stated. Moreover, these webpages had the highest proportion of mixed conceptualisations (*n* = 20, 66.67%) and the second smallest difference between continuum and categorical conceptualisations of all the webpage foci (*n* = 29, 96.67% vs. *n* = 21, 70.00%). The following statement from Beyond Blue demonstrates the mixed conceptualisations: “While we all feel sad or down from time to time, some people experience these feelings more intensely and for longer periods of time. Depression is more than just a low mood–it’s a severe condition that impacts many areas of your life.” The first statement places depression on a continuum to the universal experience of sadness. However, the second sentence emphasises that depression is categorically different to normal experiences of low mood. Of the webpages that implied a categorical conceptualisation, most of them implied a difference between depression and not having depression (*n* = 16, 76.19%), with 42.86% (*n* = 9) using medicalising language.

#### Schizophrenia Conceptualisation

Webpages focused on schizophrenia presented the second most mixed conceptualisations (*n* = 10, 62.50%). Like depression, schizophrenia webpages did not explicitly present a continuum or categorical conceptualisation. Schizophrenia webpages had the smallest difference between the proportion of continuum and categorical conceptualisations (*n* = 12, 75.00% vs. *n* = 10, 62.50%). However, all webpages presenting a continuum conceptualisation implied a continuum within schizophrenia, with only one webpage (6.25%) also supporting a continuum between schizophrenia and everyday experience. That is, they mostly talked about variations in the symptoms, severity, and duration of schizophrenia. For example, Way Ahead states: “The symptoms of disorders such as Schizophrenia may vary… They may be mild or severe.” While it appears that schizophrenia had a lower proportion of categorical presentations than depression webpages, this needs to be understood in the context of there being more unspecified schizophrenia webpages (*n* = 4, 25.00%). When excluding pages that did not present a particular conceptualisation, 83.33% of webpages implied a categorical conceptualisation. Therefore, only 6.25% (*n* = 1) of schizophrenia webpages with a conceptualisation implied a continuum from the experiences of schizophrenia to everyday experiences, whereas 83.33% implied a categorical difference between the two.

#### Conceptualisations by Population

Eleven webpages across all four foci targeted young people. All of these presented a continuum conceptualisation compared to 45.45% (*n* = 5) that also endorsed a categorical conceptualisation. The 33 webpages aimed at people with mental illness also tended to imply a continuum (*n* = 25, 75.76%) more frequently than a categorical conceptualisation (*n* = 18, 54.55%). Conceptualisation frequencies for other demographics can be found in Supplementary Materials B.

### Aetiologies

#### Mental Illness

Just over half of webpages (*n* = 13, 54.17%) listed a cause for mental illness. Further, 10 (41.67%) mentioned biological causes, 11 (45.83%) mentioned social causes, and 5 (20.83%) mentioned psychological causes, with 6 (25.00%) also mentioning substance use. Eight webpages (33.33%) mentioned either a biosocial or biopsychosocial interaction of causes.

#### Depression

Depression webpages were the most likely to mention a cause, at 86.67% (*n* = 26). There was no difference between the presentation of biological and social causes (*n* = 26, 86.67%). Depression webpages were more likely to present personality as a cause (*n* = 13, 43.33%), compared to one (4.17%) for mental illness and none for schizophrenia. Twelve webpages also presented substance use as a cause (40.00%) of depression. Thirteen webpages (43.33%) presented a biosocial or biopsychosocial interaction of causes.

#### Schizophrenia

Schizophrenia webpages had minimal difference in prevalence between biological (*n* = 12, 75.00%) and social causes (*n* = 11, 68.75%) and had the highest proportion of webpages indicating substance use as a cause (*n* = 9, 56.25%). Five pages (31.25%) provided a biosocial or biopsychosocial interaction of causes.

## Discussion

This study reviewed the prevalence of different conceptualisations and aetiologies of mental health, mental illness, depression, and schizophrenia among Australian mental health websites. Interestingly, we found that most webpages did not provide an explicit conceptualisation across any of the webpage foci under review. No webpages used explicit categorical conceptualisations and only a minority of mental health and mental illness webpages presented explicit continuum conceptualisations. Neither depression nor schizophrenia webpages used any kind of explicit conceptualisation. Therefore, the overwhelming majority of conceptualisations were implied. Furthermore, the conceptualisations for depression and schizophrenia had a sizable proportion of mixed conceptualisations, where they presented both continuum and categorical statements. Moreover, between a fifth and a half of mental health, mental illness, and schizophrenia webpages did not specify a particular conceptualisation. It is surprising that, after excluding unspecified conceptualisations, schizophrenia had the highest proportion of mixed conceptualisations, as much of the empirical research examining the effects of continuum and categorical conceptualisations on stigma focuses on schizophrenia (Schomerus et al., 2016; Thibodeau & Peterson, 2018; Wiesjahn et al., 2016). Taken together, this suggests that Australian mental health organisations may not be consciously choosing to present a particular type of conceptualisation in their online educational material. This raises concerns given that presenting different conceptualisations and aetiologies can have differential effects on mental health stigma (Kvaale et al., 2013; Schomerus et al., 2016). There is an opportunity for mental health organisations to carefully evaluate the potential impacts on stigma reduction when making decisions about how to present mental health and mental illnesses.

The higher prevalence of continuum conceptualisations than categorical conceptualisations was unexpected and at odds with prior research (Devendorf et al., 2020). This may be partly attributable to the broad coding of a continuum used in this study which included a continuum within mental health or mental illness. For instance, if the continuum-within coding was excluded then schizophrenia would have been coded as having considerably more categorical conceptualisations than continuum conceptualisations. Yet, the use of extensive criteria that distinguished between a continuum within mental illness and a continuum between mental illness and everyday experience is, to our knowledge, the first study to explore how real-world mental health promotion may present a continuum in disparate ways. Importantly, the experimental and correlational stigma literature has solely examined continuum beliefs between mental illness and everyday experience (Schomerus et al., 2016; Thibodeau & Peterson, 2018).

However, another likely explanation for the higher prevalence of continuum conceptualisations is the growing empirical evidence for continuum models of mental health and mental illness (Haslam et al., 2020; Iasellio et al., 2020). Furthermore, the continuum conceptualisation is supported by emerging research about the stigma-reduction benefits of continuum beliefs between mental illness and everyday experience (Corrigan et al., 2017; Wiesjahn et al., 2016). This research suggests that continuum explanations may be preferable to categorical ones in reducing mental health stigma (Peter et al., 2021). However, as some studies have shown either null or adverse effects (Thibodeau & Peterson, 2018), further experimental research is needed to elucidate the effect of continuum belief presentations on mental health stigma.

There were differences in the types of continuum conceptualisations presented across the four webpage foci. Both mental illness and schizophrenia had more continuum within than continuum between presentations. Conversely, mental health and depression had somewhat more continuum between than continuum within presentations. Schizophrenia was the focus with the least amount of continuum-between conceptualisations, having only one webpage comparing the experience of schizophrenia to those without schizophrenia. This aligns with research which has found that depression symptoms are more readily viewed on a continuum to everyday experience compared to schizophrenia symptoms (Angermeyer et al., 2015). Moreover, it presents an opportunity for websites to present schizophrenia symptoms as being on a continuum from mental illness to everyday experiences (e.g., perceptual disturbances when under stress being misinterpreted as occurring outside of the person) in order to reduce stigma by dissolving in-group and out-group distinctions (Link & Phelan, 2001; Peter et al., 2021). Meanwhile, there is no empirical evidence for the stigma reduction benefit of presenting a continuum within mental illness.

Only mental health and mental illness webpages presented explicit continuum conceptualisations. Some research has examined the impact of continuum belief manipulations on stigma for mental health and mental illness in general as one element within larger stigma reduction programs (Helmus et al., 2019; Szeto et al., 2019). Yet, no research has explored the impact of continuum or categorical presentations in these contexts in isolation. Thus, future research needs to explore the effect of different conceptualisations in these contexts.

Findings from our study departed from prior research (Devendorf et al., 2020) by allowing for webpages to provide a mixed conceptualisation. Given that so few webpages explicitly labelled the type of conceptualisation they were using, it is understandable that many implied both categorical and continuum conceptualisations. Moreover, presenting a categorical conceptualisation does not preclude the acknowledgement of dimensional aspects of a given phenomenon. For instance, both Boyd’s (1991) Homeostatic Property Cluster model and Niedenthal and Cantor’s (1984) account of natural categories allow for a certain degree of ‘fuzziness’ within a given category. Likewise, Zachar’s (2000) notion of practical kinds provides a way to categorise psychiatric disorders that accounts for the many variations which may be dimensional in nature. Thus, it is not altogether surprising that the proportion of mixed conceptualisations was high for schizophrenia and depression webpages, where approximately two-thirds of the conceptualisations were mixed. Our finding that mental illness/es are often conceptualised within online mental health promotion in a way that implies both continuum and categorical elements is novel. It is of particular interest as the stigma reduction literature has solely looked at the effects of presenting these conceptualisations in isolation (Schomerus et al., 2016; Violeau et al., 2020). It is unclear what the impact on stigma would be when these explanations are mixed. For instance, what would be the impact on stigma of using medicalising language alongside a continuum presentation? Future research could examine questions such as this experimentally.

It should also be noted that all webpages targeting young people presented continuum conceptualisations. This is despite there being limited research examining the link between continuum conceptualisations and stigmatising attitudes among young people aged up to 18 years (Dolphin & Hennessy, 2017; Fernandez et al., 2022). Research is needed to explore whether adult findings generalise to an adolescent population and by extension whether the widespread presentation of continuum conceptualisations is justified for this demographic. Likewise, slightly more webpages targeting people with a mental illness presented continuum rather than categorical conceptualisations. The impact of continuum belief messaging on self-stigma is unclear (Thibodeau, 2019; Violeau et al., 2020). As such, further research needs to elucidate the impact that these presentations may have on self-stigma.

In terms of aetiologies, biological and social causes were frequently mentioned across the different foci in approximately equal numbers, although the majority of webpages did not explicitly acknowledge a biosocial or biopsychosocial aetiology. Nevertheless, this shows a preference for presenting mental illness, depression, and schizophrenia as having multiple potential causes, rather than being primarily caused by biological factors as per the biogenetic view. This suggests that the biomedical model of mental illness is not particularly prevalent in Australian online mental health promotion, since the proportion of medicalising conceptualisations was also somewhat low for mental illness and depression webpages, although almost two-thirds of schizophrenia webpages were medicalising.

The mixed causal presentations evident across webpage foci may be preferable as meta-analyses indicate that presenting biological aetiologies may do more harm than good by increasing stigma (Haslam & Kvaale, 2015; Kvaale et al., 2013). Further, research suggests biopsychosocial presentations are preferable for dangerousness and unpredictability stereotypes (Walker & Read, 2002). Thus, it may be that presenting multiple causes in online mental health promotion is better than focusing on biological factors, but it is unclear how clearly recipients recognise or understand the interplay of these multiple causal factors.

### Limitations

A limitation of the present study is the moderate inter-rater reliability, particularly for the depression conceptualisation coding. The relatively poor inter-rater reliability, despite the use of clear coding criteria, may also raise questions about whether there is a lack of clarity which could be confusing to consumers. Furthermore, the use of complex criteria where webpages can be coded as presenting up to four separate conceptualisations made it more likely that coders could provide a different code for at least one of those elements. For example, even a pair of codes that agreed on three of four conceptualisation elements would be considered incorrect and subject to discussion if it differed on the final conceptualisation element (e.g., “Mixed: Explicit continuum (within/between), implicit categorical (difference) vs. Mixed: Explicit continuum (within/between), implicit categorical (difference/*medicalising*)). Moreover, as previously mentioned, depression was particularly challenging to code due to the uncertainty around whether the information met the threshold for implying a categorical conceptualisation. As such, it is more important to look at the overall trends in the conceptualisation frequencies, as there is some degree of subjectivity in determining whether a webpage meets the threshold for a given conceptualisation.

Furthermore, our review focused on Australian online mental health websites which limits the generalisability of our findings to other countries. Research shows that there are cultural differences both within and between nations regarding recognition of mental illnesses (Jorm et al., 2005b), expected treatment outcomes (Jorm et al., 2005b), stigmatising attitudes (Abdullah & Brown, 2011; Angermeyer & Dietrich, 2006; Mellor et al., 2013), and beliefs about help-seeking (Chen et al., 2020). These differences are also present among medical professionals across different countries (Richards et al., 2014; Stefanovics et al., 2016). This raises an important question, are there also cultural and national differences in how mental health and mental illness are conceptualised online? Future research is needed to explore the potential for cross-national and cultural differences in the online presentation of mental health and mental illness presentations.

## Conclusions

Overall, this study showed that continuum and categorical conceptualisations on websites could be reliably coded. Continuum conceptualisations are more prevalent than categorical conceptualisations in Australian online mental health sites. Moreover, biological and social causes are listed at about the same rate for mental illness, depression, and schizophrenia. Importantly, a substantial proportion of webpages either did not provide a conceptualisation or provided a mixed conceptualisation with statements supporting both continuum and categorical conceptualisations. Given the lack of explicit conceptualisations, there is a clear opportunity for Australian mental health organisations to actively consider how their online conceptualisations of mental health and mental illness may impact the stigma of readers.

## Supplementary Information

Below is the link to the electronic supplementary material.

Supplementary file1 (DOCX 22 KB)

Supplementary file2 (DOCX 22 KB)

Supplementary file3 (DOCX 42 KB)
