# Supplementary material for: Exploring Continuum and Categorical Conceptualisations of Mental Health and Mental Illness on Australian Websites: A Systematic Review and Content Analysis
Source: Community Ment Health J. 2022 Aug 22;59(2):275–89. doi: 10.1007/s10597-022-01005-w (PMC9859906; doi:10.1007/s10597-022-01005-w)
Supplement: Supplementary file 1 — Supplementary file1 (DOCX 22 KB) [file 10597_2022_1005_MOESM1_ESM.docx]

**Supplementary Materials A**

**Coding Criteria:**

- **Webpage Focus**
  - Mental Health
    - Focuses on mental health, as opposed to mental illness
      - E.g., “What is Mental Health?”
    - As mental health is defined differently by different websites, this section is divided further into subcategories
      - **Mental Health: Good Wellbeing**
        - Mental health is defined as referring to good wellbeing,

E.g., “Mental health is a state of good well-being where an individual is able to not only cope with everyday stresses, but to be productive and contribute to their community” (World Health Organization, 2018)

- - - - **Mental Health: General Wellbeing**
        - Mental health is defined to include all aspects of wellbeing, both good and bad

E.g., “While the majority of people usually have good mental health, anyone can experience poor mental health when faced with difficult life events.”

“Mental health can vary across our lives in the same way that physical health can. It is about our general social and emotional wellbeing.”

- - - - A page that refers to mental health as both good wellbeing and general wellbeing should be labelled “Mental Health: General Wellbeing”
    - If a page claims to be talking about ‘Mental Health’ but is primarily focused on mental illnesses, it shall be considered to have a ‘Mental Illness’ focus
  - **Mental Illness**
    - Focuses on mental illness, as opposed to mental health
    - May refer to ‘mental health conditions’, ‘mental disorders’, ‘psychiatric disorders’
      - E.g., “What is Mental Illness?”
  - **Depression**
    - Focuses on Depression or Depressive Disorders in general
      - E.g., “What is Depression?”
  - **Schizophrenia**
    - Focuses on Schizophrenia, or psychotic disorders with an emphasis on schizophrenia
      - E.g., “What is Schizophrenia?”
- **Target population**
  - - Ascertained from data itself OR ‘About us’ OR ‘Who we are’ OR ‘FAQ’ section
  - General Population
    - Mentions providing services/information to all Australians
      - E.g., “Our vision is for everyone in Australia to achieve their best possible mental health”.
  - Subgroup*
    - *Write specific subgroup identified
      - Example: Youth, Aboriginal and Torres Strait Islander, Elderly, etc.
    - Mentions providing services/information to subgroup specifically
      - E.g., “We seek to meet the needs of young people and their supports.”
  - People with Mental Illness
    - Mentions providing services/information to individuals that have a mental illness or health condition
      - E.g., “Our aim is to make a real difference in the lives of people with mental illness.”
  - Unspecified
    - Does not indicate to whom they provide services/information
- **Medicalisation**
  - **Yes**: Refers to mental illness/depression/schizophrenia as being:
    - “…a real, medical illness”
    - “…an illness like any other”
    - “Just like you can have a physical illness, you can have a mental illness”
    - “… a disease”
    - “…an illness”
      - Note: “… a mental illness/disorder” is not sufficient to be considered medicalising
  - **Somewhat**: Refers to mental illness as being:
    - “… a health problem”
    - “… a condition”
  - **No**: Description of mental illness does not fit either of the above criteria.
    - “… mental illness”
    - “… depressive illness”
    - “… psychotic illness”
    - “… mental disorder”
- **Conceptualisation**
  - **Continuum**
    - **Explicit**
      - Explicitly refers to mental health, mental illness, depression, or schizophrenia as being on a continuum/spectrum/dimensional
      - 2 types:
        - **Explicit continuum (between)**

Refers to a continuum **between** mental health and mental illness

E.g., “Mental health is a continuum with good mental health at one end and mental illness at the other”

E.g. “One way to distinguish between mental health and mental illness is to think of it as a continuum.”

E.g., “Mental illness and mental health are both on the same spectrum”

E.g., “Depression and good mental health are on the same continuum, with differences in the intensity and longevity of their experiences.”

E.g., “People without schizophrenia can relate to those with a schizophrenia diagnosis, as the symptoms of schizophrenia are on a continuum to the experiences everyday people have when facing a stressful environment.”

- - - - - **Explicit continuum (within)**

Refers to mental health as being a continuum from good mental health to poor mental health OR refers to mental illness as being a continuum from severe mental illness to mild mental illness.

Note: **Does not** mention continuum from mental health to mental illness.

E.g., “Mental health is a continuum. Individuals vary from having good mental health at one end to having poor mental health at the other”.

E.g., “People with mental illness experience symptoms on a spectrum, from severe and long-lasting experiences to mild-and short-term experiences.”

E.g., “Symptoms of depression are on a continuum from severe symptoms to mild symptoms”

E.g., “The experiences of people with schizophrenia can be placed on a continuum, from severe, chronic symptoms and dysfunction, to less frequent and milder experiences.”

- - - **Implicit**
      - Implies that mental health or mental illness are on a continuum/spectrum without explicitly stating such.
      - 2 types:
        - **Implicit continuum (between)**

Implies there is a **continuum of symptoms from people with mental illness to those without mental illness**

**OR** implies there is a continuum of experience between mental health and mental illness

E.g., “Everyone feels sad or down sometimes, especially during tough times. But, if you have these feelings intensely for long periods of time and have trouble with normal activities, you may be experiencing depression.”

E.g., “Mental health can vary from good mental health to severe mental illness.”
E.g., “While it is not uncommon for people to experience hallucinations as a response to stress, only a small subset of these people go on to develop schizophrenia”

- - - - - **Implicit continuum (within)**

Implies there is a **continuum** **within people with mental illness**. Mentions that people with mental illness can have variety of: diagnoses (e.g., MDD vs dysthymia), symptoms (e.g., poor sleep vs. weight gain), severity (e.g., mild vs severe), duration (e.g., one-off vs many episodes), outcomes (e.g., recover vs chronic).

**OR** implies there is a continuum within people’s mental health

E.g., “Mental illnesses vary both in terms of their duration and their severity”

E.g., “Everyone can experience fluctuations in their mental health throughout their lives. Sometimes our mental wellbeing may be high, other times it may be low”

E.g., “Depression can range from mild, to moderate, to severe. People with depression may experience a variety of symptoms, however not everyone experiences all of these symptoms.”

E.g., “Some people experience only one or two episodes of schizophrenia, whereas for others it may be a chronic, lifelong condition.”

- - - A webpage can imply a continuum conceptualisation through meeting both of these criteria
      - E.g., Implicit continuum (between/within)
  - **Categorical**
    - **Explicit**
      - Explicitly refers to mental illness as being categorically/fundamentally/qualitatively different to ‘normal’ functioning
        - E.g., “The experience of people with mental illness is fundamentally different to normal functioning.”
        - E.g., “People with mental illness are qualitatively different from others.”
        - E.g., “Mental illness and mental health are distinct categories”
    - **Implicit**
      - Implies that mental illness is categorically different from normal functioning without explicitly stating such. Implies a difference of ‘kind’, rather than a difference of ‘degree’.
      - There are 2 ways this conceptualisation may be implied:
        - **Implicit categorical (medicalising)**

**Medicalising mental illness** (see above criteria) by definition implies a categorical difference between having a mental illness and not having one.

‘Somewhat’ medicalising is not sufficient to be considered implicitly categorical.

E.g., “Depression is different to feeling sad or down from time to time. It is a medical illness that affects many aspects of your life.”

E.g. “Schizophrenia is a severe, medical condition.”

E.g., “Mental illnesses are brain disorders.”

- - - - - **Implicit categorical (difference)**

**Implying a qualitative difference between mental illness and mental health**

E.g., “Schizophrenia is a mental condition that disrupts the normal functioning of one’s mind”

E.g., “Depression is more than just feeling low or sad, it is a serious condition that is significantly different to normal sadness”

E.g., “Mental illness and mental health are distinctly different from one another”

Descriptions of symptoms that imply a difference from ‘normal’ population:

E.g., “Disordered thinking that can distort their perception of reality, alongside unusual ideas and extreme fears”

E.g., “The false perceptions and beliefs can result in a complete withdrawal from reality”

NOTE:

Saying a mental illness “affects how you think, feel, or behave” is not considered to be categorical.

- - - A webpage can imply a categorical conceptualisation through meeting both of these criteria
      - E.g., **Implicit categorical (difference/medicalising)**
  - **Mixed**
    - Includes statements that either implicitly or explicitly support both continuum and categorical conceptualisations
    - Indicate which conceptualisations are endorsed
      - E.g., “**Mixed: Implicit continuum (between/within); implicit categorical (medicalising)**
  - **Unspecified**
    - Does not endorse any conceptualisation of mental health or mental illness
- **Causes** 🡪 derived from ‘The Mental Illness Attribution Questionnaire’ (Knettel, 2019)
  - - Note: webpages may have multiple causes
  - **Biological**
    - Biological or medical disorder
    - Chemical imbalance in brain
    - Problems with brain function
    - Genetics
    - Family history
    - Health problems that affect biological/genetic processes
  - **Social**
    - Negative life events (e.g., death of loved one)
    - Traumatic experiences (e.g., assault)
    - Issues with social support (e.g., relationship problems)
    - Discrimination
    - Negative life stressors (e.g., financial difficulties)
    - Health problems that cause stress and other emotions tied to mental illness
  - **Psychological**
    - Negative thinking, ruminating, etc.
  - **Biopsychosocial**
    - Explicitly mentions the interplay of biological/social/psychological factors
      - E.g., “Depression is caused by the interaction of biological, environmental, and psychological causes”
  - **Biosocial**
    - Explicitly mentions the interplay of biological and social factors
      - E.g., “There are several overlapping factors that cause schizophrenia, including biology and stress.”
  - **Lifestyle**
    - Dietary problems
    - Lack of physical activity
    - Unhealthy living conditions
  - **Substance Use**
    - Drug use
    - Alcohol use
  - **Personality**
    - Personality type
  - **Personal Weakness**
    - Negative attitude
    - Lack of will or self-control
    - Personal choice to not improve
- **Health on the Net (HON) Badge**
  - Indicator of information quality
  - Can be found at the Health on the Net website
  - Presence: ‘Yes’ or ‘No’
- **Affiliation**
  - Commercial
    - Selling a product, service, or advertisement for the purpose of making a profit
  - Non-profit
    - An organisation dedicated to furthering or advocating for a social cause (e.g., stigma reduction), reinvesting revenue for this purpose
    - Has charitable status
  - University
    - A website affiliated with a particular university, usually for educational purposes
  - Government
    - A government website, denoted by .gov in domain name
  - Personal
    - A personal website
      - E.g., Blog
- **Coverage**
  - National
    - Provides information and/or services to individuals anywhere in Australia
  - State
    - Provides information and/or services to individuals within a particular state or territory
  - Local
    - Provides information and/or services to individuals within a particular town, city, or region

**References**

World Health Organization (2018). *Mental health: strengthening our response*. Retrieved May 10, 2022, from https://www.who.int/news-room/fact-sheets/detail/mental-health-strengthening-our-response
