# Supplementary material for: Exploring Continuum and Categorical Conceptualisations of Mental Health and Mental Illness on Australian Websites: A Systematic Review and Content Analysis
Source: Community Ment Health J. 2022 Aug 22;59(2):275–89. doi: 10.1007/s10597-022-01005-w (PMC9859906; doi:10.1007/s10597-022-01005-w)
Supplement: Supplementary file 2 — Supplementary file2 (DOCX 22 KB) [file 10597_2022_1005_MOESM2_ESM.docx]

**Supplementary Materials B**

*Population Frequencies across all Website Foci*

|  | General Population (*n* = 40) | People with Mental Illness^a^  (*n* = 33) | Supports^b^  (*n* = 25) | Young People  (*n* = 11) | Practitioners^c^ and Researchers  (*n* = 9) | Workers  (*n* = 5) |
| --- | --- | --- | --- | --- | --- | --- |
| Conceptualisation | Number (%) | Number (%) | Number (%) | Number (%) | Number (%) | Number (%) |
| Continuum  Implicit Continuum  Between  Within  Between/Within  Explicit Continuum  Between  Within  Between/Within  Categorical  Implicit Categorical  Difference  Medicalising  Difference/Medicalising  Explicit Categorical  Unspecified  Mixed | 29 (69.05)  28 (66.67)  1 (2.38)  19 (45.24)  8 (19.05)  3 (7.14)  3 (7.14)  0 (0.00)  0 (0.00)  19 (45.24)  19 (45.24)  4 (9.52)  7 (16.67)  8 (19.05)  0 (0.00)  11 (26.19)  17 (40.48) | 25 (75.76)  24 (72.73)  3 (9.09)  12 (36.36)  9 (27.27)  1 (3.03)  1 (3.03)  0 (0.00)  0 (0.00)  18 (54.55)  18 (54.55)  4 (12.12)  9 (27.27)  5 (15.15)  0 (0.00)  7 (21.21)  17 (51.52) | 21 (84.00)  19 (76.00)  4 (16.00)  8 (32.00)  7 (28.00)  2 (8.00)  2 (8.00)  0 (0.00)  0 (0.00)  13 (52.00)  13 (52.00)  5 (20.00)  4 (16.00)  4 (16.00)  0 (0.00)  4 (16.00)  13 (52.00) | 11 (100.00)  9 (81.82)  4 (36.36)  1 (9.09)  4 (36.36)  2 (18.18)  2 (18.18)  0 (0.00)  0 (0.00)  5 (45.45)  5 (45.45)  5 (45.45)  0 (0.00)  0 (0.00)  0 (0.00)  0 (0.00)  5 (45.45) | 7 (70.00)  7 (70.00)  1 (10.00)  1 (10.00)  5 (50.00)  0 (0.00)  0 (0.00)  0 (0.00)  0 (0.00)  4 (40.00)  4 (40.00)  2 (20.00)  1 (10.00)  1 (10.00)  0 (0.00)  3 (30.00)  4 (40.00) | 5 (100.00)  3 (60.00)  1 (20.00)  2 (40.00)  0 (0.00)  3 (60.00)  2 (40.00)  1 (20.00)  0 (0.00)  2 (40.00)  2 (40.00)  2 (40.00)  0 (0.00)  0 (0.00)  0 (0.00)  0 (0.00)  2 (40.00) |

^a^Webpages focused on people with health conditions were included as mental illness was considered a health condition on these websites. ^b^Includes parents, families, and other types of supports. ^c^Includes health and mental health workers. Note: Some webpages focused on multiple populations (e.g., general population and people with mental illness), in which case they were included in both population columns. Only populations with *n* > 5 were included. The same website can appear multiple times due to having different webpages for different foci.
