# Supplementary material for: Exploring Continuum and Categorical Conceptualisations of Mental Health and Mental Illness on Australian Websites: A Systematic Review and Content Analysis
Source: Community Ment Health J. 2022 Aug 22;59(2):275–89. doi: 10.1007/s10597-022-01005-w (PMC9859906; doi:10.1007/s10597-022-01005-w)
Supplement: Supplementary file 3 — Supplementary file3 (DOCX 42 KB) [file 10597_2022_1005_MOESM3_ESM.docx]

**Supplementary Materials C – Webpage Data**

Table 1. *Mental Health Webpages*

| Website | Focus | Population | Conceptualisation | Affiliation | HON^ | Coverage |
| --- | --- | --- | --- | --- | --- | --- |
| Australia Counselling | Good wellbeing | General population | Unspecified | Commercial | No | National |
| Australian Indigenous HealthInfoNet | General wellbeing | Aboriginal and Torres Strait Islanders, practitioners and policymakers | Unspecified | University | Yes | National |
| Beyond Blue | Good wellbeing | General population, people with mental illness | Explicit continuum (between) | Non-profit | No | National |
| Black Dog Institute | Good wellbeing | General population | Unspecified | Non-profit | Yes | National |
| Everymind | Good wellbeing | General population | Unspecified | Non-profit | No | National |
| Head to Health | Good wellbeing | General population, people with mental health difficulties and their supports, health professionals | Unspecified | Government | No | National |
| Heads Up | Good wellbeing | Employers, employees, businesses | Explicit continuum (between) | Non-profit | No | National |
| Headspace | Good wellbeing | Young people, people supporting young people | Mixed: Explicit continuum (between), implicit categorical (difference) | Non-profit | No | National |
| Health.gov.au | Good wellbeing | General population | Implicit continuum (within) | Government | No | National |
| Healthdirect | Good wellbeing | General population | Unspecified | Government | Yes | National |
| Healthy WA | Good wellbeing | General population, people with health or medical conditions | Unspecified | Government | No | State |
| Mental Health Australia | Good wellbeing | General population | Unspecified | Non-profit | No | National |
| Mental Health Online | General wellbeing | People with mental illness | Implicit continuum (between) | University | No | National |
| NIB Health Insurance | Good/general wellbeing | General population | Implicit continuum (within) | Commercial | No | National |
| NSW Health | Good wellbeing | Unspecified | Unspecified | Government | No | State |
| Reach Out | General wellbeing | Young people and their parents | Implicit continuum (between) | Non-profit | No | National |
| SANE | Good wellbeing | People with mental illness and their supports | Implicit continuum (between) | Non-profit | Yes | National |
| Sydney North Health Network | Good wellbeing | Health patients | Unspecified | Government | No | Local |
| The University of Sydney | Good wellbeing | University students | Unspecified | University | No | Local |
| The University of Western Australia | General wellbeing | University staff and students | Explicit continuum (within) | University | No | Local |
| Way Ahead | General wellbeing | General population | Implicit continuum (within) | Non-profit | No | State |
| Western Australia Mental Health Commission | Good Wellbeing | General population | Explicit continuum (between), implicit continuum (within) | Government | No | State |

^Health on the Net badge.

Table 2. *Mental Illness Webpages*

| Website | Population | Conceptualisation | Medicalisation | Causes | Affiliation | HON^ | Coverage |
| --- | --- | --- | --- | --- | --- | --- | --- |
| Australia Counselling | General population | Unspecified | No | Biological, social, psychological, biopsychosocial | Commercial | No | National |
| Better Health Channel (Victoria) | People with health and medical conditions | Implicit categorical (medicalising) | Yes | Social | Government | Yes | State |
| Black Dog Institute | General population | Unspecified | No | Unspecified | Non-profit | Yes | National |
| Everymind | General population | Implicit continuum (within) | Somewhat | Social | Non-profit | No | National |
| Head to Health | General population, people with mental health difficulties and their supports, health professionals | Implicit continuum (within) | No | Unspecified | Government | No | National |
| Heads Up | Employers, employees, businesses | Explicit continuum (between), implicit continuum (within) | Somewhat | Unspecified | Non-profit | No | National |
| Headspace | Young people, people supporting young people | Mixed: Explicit continuum (between), implicit categorical (difference) | No | Biological, social, psychological, biopsychosocial | Non-profit | No | National |
| Health.gov.au | General population | Implicit continuum (within) | No | Unspecified | Government | No | National |
| Healthdirect | General population | Mixed: Implicit continuum (within), implicit categorical (medicalising) | Yes | Biological, social, biosocial, substance use, personality | Government | Yes | National |
| HealthyWA | General population, people with health or medical conditions | Mixed: Implicit continuum (within), implicit categorical (medicalising) | Yes | Biological, social, psychological, biopsychosocial, substance use | Government | No | State |
| Mental Health Australia | General population | Implicit categorical (medicalising) | Yes | Unspecified | Non-profit | No | National |
| Mental Health Foundation Australia | General population | Mixed: Implicit continuum (within), implicit categorical (difference/medicalising) | Yes | Biological | Non-profit | No | National |
| Mind Australia | People with mental illness and their supports | Unspecified | Somewhat | Unspecified | Non-profit | No | National |
| Mission Australia | General population | Implicit continuum (within) | No | Unspecified | Non-profit | No | National |
| NIB Health Insurance | General population | Mixed: Implicit continuum (between), implicit categorical (difference) | No | Unspecified | Commercial | No | National |
| NSW Health | Unspecified | Mixed: Implicit continuum (within), implicit categorical (medicalising) | Yes | Unspecified | Government | No | State |
| One Door Mental Health | People with mental illness and their families | Unspecified | Somewhat | Biological, social, biosocial | Non-profit | No | National |
| Reach Out | Young people and their parents | Implicit continuum (between) | No | Biological, social, psychological, biopsychosocial, substance use | Non-profit | No | National |
| SANE | People with mental illness and their supports | Mixed: Implicit continuum (within), implicit categorical (medicalising) | Yes | Biological, social, biosocial, substance use, lifestyle | Non-profit | Yes | National |
| SANE | Young people | Implicit continuum (between) | No | Substance use | Non-profit | Yes | National |
| Sydney Local Health District | Patients and their families | Mixed: Implicit continuum (between/within), implicit categorical (medicalising) | Yes | Biological, social | Government | No | Local |
| Sydney North Health Network | Health patients | Implicit continuum (within) | Somewhat | Unspecified | Government | No | Local |
| Way Ahead | General population | Unspecified | Somewhat | Unspecified | Non-profit | No | State |
| Western Australia Mental Health Commission | General population | Explicit continuum (between), implicit continuum (within) | No | Biological, social, psychological, biopsychosocial, substance use, lifestyle | Government | No | State |

^Health on the Net badge.

Table 3. *Depression Webpages*

| Website | Population | Conceptualisation | Medicalisation | Causes | Affiliation | HON^ | Coverage |
| --- | --- | --- | --- | --- | --- | --- | --- |
| Australian Indigenous HealthInfoNet | Aboriginal and Torres Strait Islanders, practitioners and policymakers | Implicit continuum (between/within) | No | Biological, social, personality, substance use | University | Yes | National |
| Australian Psychological Society | Psychologists, general population | Mixed: Implicit continuum (between), implicit categorical (difference) | No | Biological, social, psychological, biopsychosocial | Non-profit | No | National |
| Better Health Channel (Victoria) | People with health and medical conditions | Mixed: Implicit continuum (between/within), implicit categorical (difference) | Somewhat | Biological, social, psychological, biopsychosocial, personality, substance use, lifestyle | Government | Yes | State |
| Beyond Blue | General population, people with mental illness | Mixed: Implicit continuum (between/within), implicit categorical (difference) | Somewhat | Biological, social, biosocial, personality, substance use | Non-profit | No | National |
| Black Dog Institute | General population | Mixed: Implicit continuum (between/within), implicit categorical (difference/medicalising) | Yes | Biological, social, biosocial, personality | Non-profit | Yes | National |
| Black Dog Institute | Young people | Implicit continuum (between) | No | Biological, social | Non-profit | Yes | National |
| Black Dog Institute | Older people | Implicit continuum (between) | No | Biological, social, personality, lifestyle | Non-profit | Yes | National |
| Centre for Clinical Interventions | People with health and medical conditions, health professionals | Mixed: Implicit continuum (between/within), implicit categorical (medicalising) | Yes | Biological, social, psychological, biopsychosocial | Government | No | State |
| Diabetes Australia | General population, people with or at risk of developing diabetes | Implicit categorical (difference/medicalising) | Yes | Biological, social | Non-profit | No | National |
| Heads Up | Employers, employees, businesses | Mixed: Implicit continuum (between), implicit categorical (difference) | Somewhat | Unspecified | Non-profit | No | National |
| Headspace | Young people, people supporting young people | Mixed: Implicit continuum (between/within), implicit categorical (difference) | No | Biological, social, lifestyle, substance use | Non-profit | No | National |
| Head to Health | General population, people with mental health difficulties and their supports, health professionals | Implicit continuum (between/within) | No | Unspecified | Government | No | National |
| Healthdirect | General population | Implicit continuum (between/within) | Somewhat | Biological, social, biosocial, personality, substance use, lifestyle | Government | Yes | National |
| Health.gov.au | General population | Mixed: Implicit continuum (between/within), implicit categorical (medicalising) | Yes | Biological, social, psychological, biopsychosocial, personality, substance use | Government | No | National |
| Kids Helpline | Young people | Mixed: Implicit continuum (between/within), implicit categorical (difference) | No | Biological, social, psychological, personality | Non-profit | No | National |
| Lifeline | General population | Mixed: Implicit continuum (between/within), implicit categorical (difference) | No | Biological, social, biosocial, personality, substance use | Non-profit | No | National |
| Mensline | Men | Mixed: Implicit continuum (between/within), implicit categorical (medicalising) | Yes | Biological, social, psychological | Non-profit | No | National |
| Mensline | Older men | Implicit continuum (between) | No | Biological, social, psychological | Non-profit | No | National |
| Mind Australia | People with mental illness and their supports | Mixed: Implicit continuum (between), implicit categorical (difference) | No | Biological, social | Non-profit | No | National |
| NeuRA | General population, people with brain or mind disorders | Mixed: Implicit continuum (within), implicit categorical (difference) | No | Biological, social | Non-profit | No | National |
| NSW Health | Unspecified | Implicit continuum (within) | No | Unspecified | Government | No | State |
| Open Arms | Current and ex-serving Australian Defence Force personnel and their families | Mixed: Implicit continuum (within), implicit categorical (difference) | Somewhat | Biological, social, biosocial | Government | No | National |
| Orygen | Young people, health professionals | Mixed: Implicit continuum (between/within), implicit categorical (difference) | No | Biological, social, personality, substance use | Non-profit | No | National |
| Reach Out | Young people and their parents | Implicit continuum (between/within) | Somewhat | Biological, social, biosocial | Non-profit | No | National |
| SANE | People with mental illness and their supports | Mixed: Implicit continuum (between/within), implicit categorical (difference/medicalising) | Yes | Biological, social, biosocial | Non-profit | Yes | National |
| Sydney Local Health District | Patients and their families | Mixed: Implicit continuum (between/within), implicit categorical (medicalising) | Yes | Biological, social psychological, biopsychosocial, personality, substance use | Government | No | Local |
| The University of Sydney | University students | Mixed: Implicit continuum (between), implicit categorical (difference) | No | Unspecified | University | No | Local |
| This Way Up | People with mental illness | Implicit continuum (between/within) | No | Biological, social, personality, substance use | Non-profit | No | National |
| Way Ahead | General population | Mixed: Implicit continuum (within), implicit categorical (difference/medicalising) | Yes | Biological, social, psychological, biopsychosocial, personality, substance use | Non-profit | No | State |
| Your Health in Mind | General population | Mixed: Implicit continuum (between/within), implicit categorical (medicalising) | Yes | Biological, social, substance use, lifestyle | Medical College | No | National |

^Health on the Net badge.

Table 4. *Schizophrenia Webpages*

| Website | Population | Conceptualisation | Medicalisation | Causes | Affiliation | HON^ | Coverage |
| --- | --- | --- | --- | --- | --- | --- | --- |
| Better Health Channel (Victoria) | People with health and medical conditions | Mixed: Implicit continuum (within), implicit categorical (difference/medicalising) | Yes | Biological | Government | Yes | State |
| Garvan Institute of Medical Research | Patients and researchers | Unspecified | No | Biological, social, substance use | Non-profit | No | National |
| Head to Health | General population, people with mental health difficulties and their supports, health professionals | Mixed: Implicit continuum (between/within), implicit categorical (difference/medicalising) | Yes | Substance use | Government | No | National |
| Health Engine | Patients | Mixed: implicit continuum (within), implicit categorical (medicalising) | Yes | Biological, social, substance use | Commercial | No | National |
| Healthdirect | General population | Mixed: Implicit continuum (within), implicit categorical (difference/medicalising) | Yes | Biological, social, biosocial, substance use, lifestyle | Government | Yes | National |
| Healthy WA | General population, people with health or medical conditions | Mixed: Implicit continuum (within), implicit categorical (medicalising) | Yes | Biological, social, biosocial, substance use, lifestyle | Government | No | State |
| Helping Minds | Carers, friends and family of people with schizophrenia | Unspecified | No | Biological, social, biosocial | Non-profit | No | National |
| Mind Australia | People with mental illness and their supports | Mixed: Implicit continuum (within), implicit categorical (medicalising) | Yes | Unspecified | Non-profit | No | National |
| NeuRA | General population, people with brain or mind disorders | Unspecified | No | Biological, social, biosocial | Non-profit | No | National |
| NSW Health | Unspecified | Unspecified | No | Unspecified | Government | No | State |
| One Door Mental Health | People with mental illness and their families | Mixed: Implicit continuum (within), implicit categorical (difference/medicalising) | Yes | Unspecified | Non-profit | No | National |
| Reach Out | Young people and their parents | Implicit continuum (within) | No | Biological, social, substance use | Non-profit | No | National |
| SANE | People with mental illness and their supports | Implicit continuum (within) | No | Biological, social, substance use | Non-profit | Yes | National |
| Sydney Local Health District | Patients and their families | Mixed: Implicit continuum (within; implicit categorical (difference/medicalising) | Yes | Biological, social, substance use | Government | No | National |
| Way Ahead | General population | Mixed: Implicit continuum (within), implicit categorical (difference/medicalising) | Yes | Biological, social, psychological, biopsychosocial | Non-profit | No | State |
| Your Health in Mind | General population | Mixed: Implicit continuum (within), implicit categorical (difference/medicalising) | Yes | Biological, social, substance use | Medical College | No | National |

^Health on the Net badge
